# Days at home after surgery as a perioperative outcome: scoping review and recommendations for use in health services research

**DOI:** 10.1093/bjs/znae278

**Published:** 2024-12-04

**Authors:** Tiago Ribeiro, Armaan K Malhotra, Adom Bondzi-Simpson, Antoine Eskander, Negar Ahmadi, Frances C Wright, Daniel I McIsaac, Alyson Mahar, Angela Jerath, Natalie Coburn, Julie Hallet

**Affiliations:** Department of Surgery, University of Toronto, Toronto, Ontario, Canada; Institute of Health Policy Management and Evaluation, University of Toronto, Toronto, Ontario, Canada; Department of Surgery, University of Toronto, Toronto, Ontario, Canada; Institute of Health Policy Management and Evaluation, University of Toronto, Toronto, Ontario, Canada; Department of Surgery, University of Toronto, Toronto, Ontario, Canada; Institute of Health Policy Management and Evaluation, University of Toronto, Toronto, Ontario, Canada; Institute of Health Policy Management and Evaluation, University of Toronto, Toronto, Ontario, Canada; Clinical Evaluative Sciences, Sunnybrook Research Institute, Toronto, Ontario, Canada; Division of Surgical Oncology, Odette Cancer Centre—Sunnybrook Health Sciences Centre, Toronto, Ontario, Canada; Department of Surgery, University of Toronto, Toronto, Ontario, Canada; Department of Surgery, University of Toronto, Toronto, Ontario, Canada; Institute of Health Policy Management and Evaluation, University of Toronto, Toronto, Ontario, Canada; Division of Surgical Oncology, Odette Cancer Centre—Sunnybrook Health Sciences Centre, Toronto, Ontario, Canada; Department of Anesthesiology and Pain Medicine, University of Ottawa, Ottawa, Ontario, Canada; School of Epidemiology and Public Health, University of Ottawa, Ottawa, Ontario, Canada; The Ottawa Hospital Research Institute, Ottawa, Ontario, Canada; School of Nursing, Queen’s University, Kingston, Ontario, Canada; Institute of Health Policy Management and Evaluation, University of Toronto, Toronto, Ontario, Canada; Clinical Evaluative Sciences, Sunnybrook Research Institute, Toronto, Ontario, Canada; Department of Anesthesiology and Pain Medicine, University of Toronto, Toronto, Ontario, Canada; Department of Anesthesia, Sunnybrook Health Sciences Centre, Toronto, Ontario, Canada; Department of Surgery, University of Toronto, Toronto, Ontario, Canada; Institute of Health Policy Management and Evaluation, University of Toronto, Toronto, Ontario, Canada; Clinical Evaluative Sciences, Sunnybrook Research Institute, Toronto, Ontario, Canada; Division of Surgical Oncology, Odette Cancer Centre—Sunnybrook Health Sciences Centre, Toronto, Ontario, Canada; Department of Surgery, University of Toronto, Toronto, Ontario, Canada; Institute of Health Policy Management and Evaluation, University of Toronto, Toronto, Ontario, Canada; Clinical Evaluative Sciences, Sunnybrook Research Institute, Toronto, Ontario, Canada; Division of Surgical Oncology, Odette Cancer Centre—Sunnybrook Health Sciences Centre, Toronto, Ontario, Canada

## Abstract

**Background:**

Days at home after surgery is a promising new patient-centred outcome metric that measures time spent outside of healthcare institutions and mortality. The aim of this scoping review was to synthesize the use of days at home in perioperative research and evaluate how it has been termed, defined, and validated, with a view to inform future use.

**Methods:**

The search was run on MEDLINE, Embase, and Scopus on 30 March 2023 to capture all perioperative research where days at home or equivalent was measured. Days at home was defined as any outcome where time spent outside of hospitals and/or healthcare institutions was calculated.

**Results:**

A total of 78 articles were included. Days at home has been increasingly used, with most studies published in 2022 (35, 45%). Days at home has been applied in multiple study design types, with varying terminology applied. There is variability in how days at home has been defined, with variation in measures of healthcare utilization incorporated across studies. Poor reporting was noted, with 14 studies (18%) not defining how days at home was operationalized and 18 studies (23%) not reporting how death was handled. Construct and criterion validity were demonstrated across seven validation studies in different surgical populations.

**Conclusion:**

Days at home after surgery is a robust, flexible, and validated outcome measure that is being increasingly used as a patient-centred metric after surgery. With growing use, there is also growing variability in terms used, definitions applied, and reporting standards. This review summarizes these findings to work towards coordinating and standardizing the use of days at home after surgery as a patient-centred policy and research tool.

## Teaser

A scoping review was performed to evaluate the use of days at home in perioperative health services research, finding increasing use across multiple patient populations and study designs, with significant heterogeneity in terminology, definitions, analysis, and reporting. With the aim of improving and consolidating future use, best practice recommendations are provided for use of days at home.

## Introduction

The optimal choice of metric to evaluate outcomes in surgery is an area of ongoing study. Increasingly, traditional endpoints, such as morbidity, length of stay, and mortality, are recognized as not independently capturing all aspects of care relevant to patients^1,2^. As a result, there has been increasing interest in defining outcomes that are patient-centred and valid in surgical quality-of-care research^2–4^.

In view of standardizing patient-centred endpoints in surgical trials, the Standardised Endpoints in Perioperative Medicine (StEP) group has defined recommended endpoints through a consensus Delphi process of international experts, informed by a systematic review of existing ouctomes^5^. They recommended the novel patient-centred outcome ‘days at home’ (DAH) after surgery as an optimal endpoint to capture life impact^5^. DAH is a composite measure that represents the time a patient spends alive and out of hospitals and institutions. It captures the overall burden of morbidity, length of stay, functional impact, and recovery on a patient’s life in a simple and easily summarized metric. In accordance with the recommendations from the StEP group, DAH is being increasingly used as a patient-centred outcome in surgical observational research and clinical trials^6–8^.

The outcome DAH was introduced in the early 2000s in the cardiology literature and has more recently grown in use as a perioperative outcome metric^1,3,4,6–13^. Given its recent introduction into the surgical literature, it is hypothesized that there is heterogeneity in its use, terminology, and definitions. As a result, broad understanding of this new metric is limited, which may hinder knowledge dissemination and use. Therefore, the aim of this scoping review was to map the literature on DAH after surgery, review definitions, and consolidate validation studies. This work was conducted with a view to guide future use of DAH in perioperative research, clinical decision-making, and policy.

## Methods

This scoping review was designed and conducted using joint guidance from the *JBI Manual for Evidence Synthesis*^14^ and the expanded framework of Arksey and O’Malley^10,11^. A previously published protocol on the use of DAH in cancer care was adapted for this review^12^. This scoping review adheres to the PRISMA extension for scoping reviews^13,14^. It has been registered with the Open Science Framework (https://osf.io/m4tqz/).

### Objectives

This scoping review aimed to answer the following research questions:

- In what context has DAH been used as an outcome measure in perioperative research?
- How has DAH been termed and defined as an outcome in perioperative research?
- Has DAH been validated in perioperative research settings?

### Search and eligibility

All studies measuring DAH after surgery in adults (age greater than or equal to 18 years) were included. Studies measuring DAH after endoscopic procedures alone were excluded. DAH was defined as a composite outcome, recording time a patient spends alive and out of hospitals and/or healthcare institutions. This definition sought to include all applications of any measure that used healthcare utilization time to capture overall surgical impact. Healthcare institutions included emergency departments, rehabilitation centres, long-term care homes, and other healthcare facilities. The use of the term DAH in this review refers to all variables measuring the same concept, acknowledging the variability in terminology and definitions used. Studies measuring the inverse of DAH, such as ‘institution days’, were also captured. Randomized and non-randomized trials, prospective and retrospective cohort studies, case–control studies, cross-sectional studies, quasi-experimental studies, and case series of ten or more participants were included. There were no publication year restrictions. Grey literature was excluded. All geographical regions and languages were included; however, the literature search was only performed in English. Inclusion and exclusion criteria are further detailed in *Table 1*.

**Table 1:** Inclusion and exclusion criteria

	Inclusion criteria	Exclusion criteria
Outcome	Measurement of outcome ‘days at home’ or equivalent*	No measurement of outcome ‘days at home’ or equivalent*
Exposure	Any surgical interventions	No surgical intervention Endoscopic interventions only
Population	Age ≥18 years Surgical intervention	Age <18 years
Study details	Randomized and non-randomized trials Prospective and retrospective cohort studies Case–control studies Cross-sectional studies Quasi-experimental studies Case series of ten or more participants All geographical regions and languages	Editorials, opinion pieces, case reports, dissertations, conference abstracts, and protocols Reviews and narrative studies Grey literature

^*^Defined as a composite outcome, recording time a patient spends alive and out of hospitals and institutions. Inverse measures, recording time spent in hospitals and institutions, also included.

### Search strategy and information sources

The search strategy was developed for MEDLINE and adapted to Embase and Scopus through consultation with a librarian at the University of Toronto. A list of text words was chosen based on a preliminary literature review. Additional terms were added to the search in an iterative manner as reviewers explored the evidence base (*Tables S1*–*S3*). The final search was run on 30 March 2023.

### Study selection

An initial pilot testing phase of eligibility criteria was completed on a random sample of 25 titles and abstracts by two independent reviewers until inter-rater reliability of over 75% was achieved. The first stage of study selection involved title and abstract examination based on inclusion and exclusion criteria by two independent reviewers. The second stage involved full-text review by two independent reviewers. Disagreements during both stages were addressed through reviewer consensus or by consultation with the research team. Management of search results was completed using Covidence systematic review software (Veritas Health Innovation, Melbourne, Victoria, Australia).

### Data extraction and analysis

Data extraction followed an iterative process as outlined by expert guidelines, with updates to tables as deemed necessary by the research team^11,14,15^. Key study characteristics extracted included study design, study aim, surgical specialty, patient population, methodological components, exposure or comparators, definition of DAH, and main findings. Surgical specialty categories for data extraction were selected based on the included studies. Publications were divided into three study types based on their study aims: descriptive, effectiveness, and validation studies. Descriptive studies were those that aimed to describe DAH in a study population without the evaluation of a causal relationship. Effectiveness studies (including both observational research and clinical trials) were those that aimed to evaluate the causal effect of one exposure on DAH. Validation studies were those that aimed to validate DAH in their study population. Data analysis involved a descriptive evaluation of key study characteristics and a thematic analysis^11,14^. Thematic analysis centred around study types and rationale for use of the outcome DAH.

## Results

### Sources of evidence

**Fig. 1:**
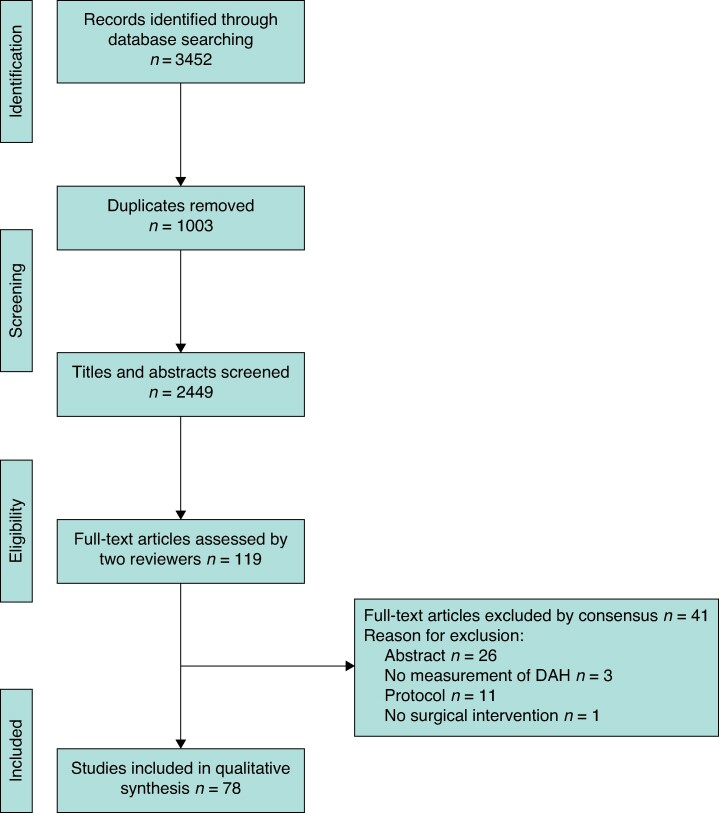
PRISMA study selection flow diagram DAH, days at home.

A total of 2449 abstracts were screened, from which 119 full-text articles were reviewed, leading to 78 articles being included in the narrative synthesis. The most common reason for study exclusion was abstract-only publication (26, 63.4%). See *Fig. 1* for the PRISMA flow diagram.

### Study characteristics

**Fig. 2:**
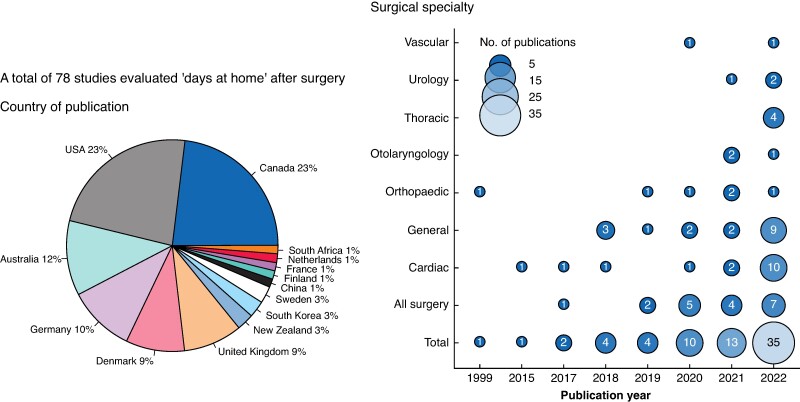
Characteristics of studies evaluating days at home after surgery A total of eight publications (published between January and March 2023) have been omitted from the right-hand chart. Cardiac surgery includes cardiac transplant surgery. General surgery includes colorectal, hepatobiliary, bariatric, and abdominal transplant surgery. Orthopaedic surgery includes spine surgery.

There were 14 countries where DAH has been evaluated in perioperative research, with most studies performed in Canada (18, 23.1%) and the USA (18, 23.1%). The first study measuring DAH after surgery was published in 1999 as a measure of quality of life in patients who underwent surgery for symptomatic spinal metastasis^16^. There were no publications thereafter until 2005, followed by an increasing number of studies until 2022, when 35 studies were published. The surgical disciplines with the most studies evaluating DAH were cardiac surgery (20, 25.6%) and general surgery (20, 25.6%). In total, seven different surgical disciplines have evaluated DAH (*Fig. 2*). Of the 78 studies, there were 55 unique corresponding authors. Most studies were descriptive (54, 69.2%), followed by effectiveness studies (18, 23.1%), and validation studies (6, 7.7%). The majority of studies were retrospective cohorts (58, 74.4%)^6–8,16–70^, followed by prospective cohorts (10, 12.8%)^71–80^, clinical trials (6, 7.7%)^81–86^, secondary analyses of trial data (3, 3.8%)^87–89^, and a single quasi-experimental study (1.3%)^90^. Across the included studies DAH was conceptualized as a patient-centred metric capturing a surrogate of quality of life. A summary of the data extracted is available in *Table S4*.

### Terms and definitions of days at home after surgery

**Fig. 3:**
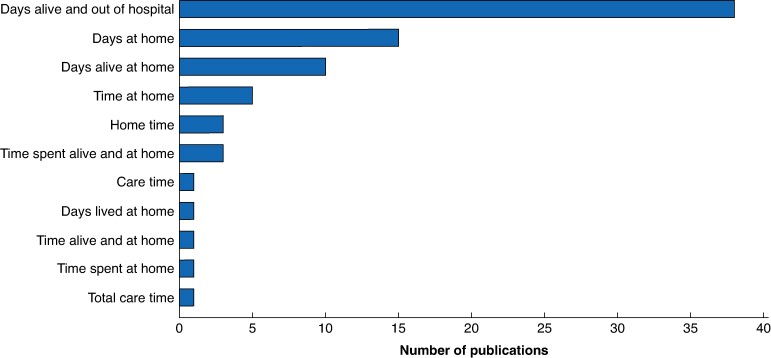
Terms used for ‘days at home’ after surgery in perioperative research

**Fig. 4:**
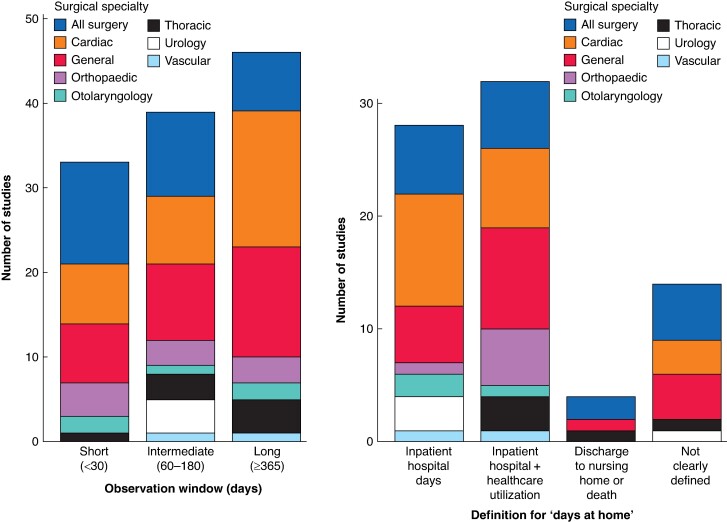
Technical aspects in the use of days at home after surgery: observation window and definitions applied Healthcare utilization includes a combination of days in nursing units, long-term care, and rehabilitation centres, as well as outpatient visits, commute time, and time receiving outpatient treatments, depending on study.

There were 12 unique terms used to represent the composite measure DAH after surgery. The most common terms were ‘days alive and out of hospital’ (38, 48.1%), followed by ‘days at home’ (15, 19.0%) (*Fig. 3*). The definition of DAH varied across studies by incorporating different measures of healthcare utilization and having a different observation window (*Fig. 4*). The most common definition incorporated both inpatient hospital days and other healthcare institution days, including days spent in rehabilitation, long-term care, nursing homes, and other institutions (32, 40.5%). Within this group there was variability, with the most comprehensive definition of DAH incorporating time in receipt of outpatient treatments, commute time, and outpatient visits, in addition to days in institution^44,80^. The least comprehensive definition included inpatient hospital days after surgery in the postoperative observation window (29, 36.7%). In these two cases, calculation would be as follows: (total days observed) − (healthcare utilization days). Of the remaining studies, 4 (5.1%) operationalized DAH as the number of days after surgery until nursing home admission or death and 14 (17.7%) did not clearly define how DAH was calculated. The postoperative observation window varied, with multiple studies including more than one observation window in their calculation of DAH (*Fig. 4*). Handling of death in the calculation of DAH was variable across studies. The most common method was to handle dead days as institution days (28, 35.9%). Other methods included handling death as a DAH of zero (16, 20.5%), censoring observations (15, 19.2%), and excluding patients (1, 1.3%). A total of 18 studies (23.1%) did not report how patient deaths were handled.

### Analysis of days at home after surgery

The distribution of DAH after surgery was consistent across the included studies. DAH was heavily left skewed, with a spike at zero across different populations and observation windows. The relative proportion of zeros was dependent on the population studied and how death was handled. A total of 48 studies (61.5%) performed multivariable regression; of these, 2 (4.2%) re-operationalized DAH as a binary and used logistic regression^34,48^ and 8 (16.7%) re-operationalized DAH in a time-to-event analysis^6,7,20,30–33,69^. The remaining studies that employed a multivariable analysis used quantile regression (17, 35.4%)^17,19,36–38,40,44,45,49,53,59,61,63,76,77,88,89^, linear regression (10, 20.8%),^21,22,45,47,58,60,62,74,79,82^, Poisson regression (7, 14.6%)^24,25,42,43,65,84,85^, negative binomial regression (4, 8.3%)^18,33,50,64^, or β regression (1, 2.1%)^70^. Other methods employed for confounder adjustment were instrumental variable analysis (2, 2.6%)^27,35^ and propensity score methods (5, 6.4%)^24,33,57,63,68^. Of the included studies, two studies (4.2%) commented on or explored the minimally important difference in DAH. This was reported either as 1 day based on clinical rationale^59^ or 11 days over DAH-90 based on anchor-based and distribution-based methods^49^.

### Validation of days at home after surgery

A total of seven studies have validated DAH after surgery^8,36,46,49,59,66,89^. These included both heterogeneous patient population studies and procedure-specific validation studies (*Table 2*). Construct validity was demonstrated in all these studies by observing DAH was associated with patient-, surgery-, and system-level factors known to be related to outcomes after surgery. In addition, criterion validity, including predictive validity, was demonstrated by observing DAH could predict or was associated with postoperative complications, 1-year survival, or overall mortality^8,36,46,49,59,66,89^.

**Table 2:** Surgical validation studies of days at home after surgery

Study	Country	Study design	Surgical population	Outcome term: definition*	Construct validity†: associations	Criterion validity‡: associations	Notes
Bell *et al*.^8^	Sweden	Multicentre registry retrospective cohort study	Adults aged ≥18 years undergoing surgery (*n* = 1 125 434)	‘Days at home’ over 30 or 90 days: calculated using index admission and readmission. Mortality DAH = 0	Age Co-morbidities Operative approach Operating time Sex Surgery type	Complications One-year mortality	Increased statistical efficiency compared with mortality and complications. Increased predictive utility compared with length of stay.
Jerath *et al*.^36^	Canada	Provincial retrospective cohort study	Adults aged ≥40 years undergoing defined elective high- and intermediate-risk non-cardiac surgical procedures (*n* = 540 072)	‘Days alive and out of hospital’ at 30, 90, or 180 days: calculated using index admission and readmission. Mortality DAH = 0	Age Co-morbidities Operating time Operative approach Sex Surgery type	DAH-90 DAH-180 Complciations Mortality	DAH over 30 days was strongly associated with DAH-90 and DAH-180.
McIsaac *et al*.^49^	Canada	Population-based retrospective cohort study	Adults aged ≥50 years with hip fracture admission (*n* = 63 778)	‘Days alive and at home’ at 90 days: calculated using index admission, readmission, long-term care, and respite. Mortality DAH = 0 Secondary: 360 days	Age Co-morbidities Frailty Hospital volume Sex	Complications DAH-365 Mortality	Findings were robust to sensitivity analysis treating long-term care as home. Minimally important difference of 11 days.
M’Pembele *et al*.^46^	Germany	Single-centre retrospective cohort study	Adults aged ≥18 years undergoing heart transplant (*n* = 175)	‘Days alive and out of hospital’ at 1 year: calculated using index admission, readmission, rehabilitation, and other institutions. Dead days = institution days	Co-morbidities	Complications	Findings robust to sensitivity analysis excluding mortality.
Myles *et al*.^89^	Australia	Secondary analysis of multicentre trial data	Adults aged ≥18 years undergoing surgery (*n* = 2109)	‘Days at home’ over 30 days: calculated using index admission and readmission. Mortality DAH = 0	Age Co-morbidities Operating time Poor functional status Smoking status	Complications	Findings robust to sensitivity analysis performed using estimates of rehabilitation days.
Reilly *et al*.^59^	Australia	Private health insurer data retrospective cohort study	Adults aged ≥18 years undergoing surgery (*n* = 181 281)	‘Days alive and at home’ 30 days after surgery: calculated using index admission, readmission, and nursing home. Mortality DAH = 0	Admission type Age Co-morbidities	Complications	Increase in DAH was associated with decreased total hospital cost.
Spurling *et al*.^66^	UK	Multicentre registry retrospective cohort study	Adults aged ≥18 years undergoing emergency laparotomy (*n* = 78 921)	‘Days alive and out of hospital’ at 30, 90, 180, or 365 days: calculated using index admission and readmission. Mortality DAH = 0	Admission type Age Co-morbidities Operative approach	One-year mortality	–

^*^All outcomes calculated from date of index surgery. †Construct validity: extent by which DAH accurately measures construct of postoperative outcomes through expected associations with known predictors of poor outcomes, including patient-, surgery-, and system-level factors. Associations of multivariable analyses displayed when available. ‡Criterion validity (includes predictive validity): extent by which DAH can predict or correlate with other known postoperative outcomes. DAH, days at home; DAH-*x* (where *x* is a number).

## Discussion

**Fig. 5:**
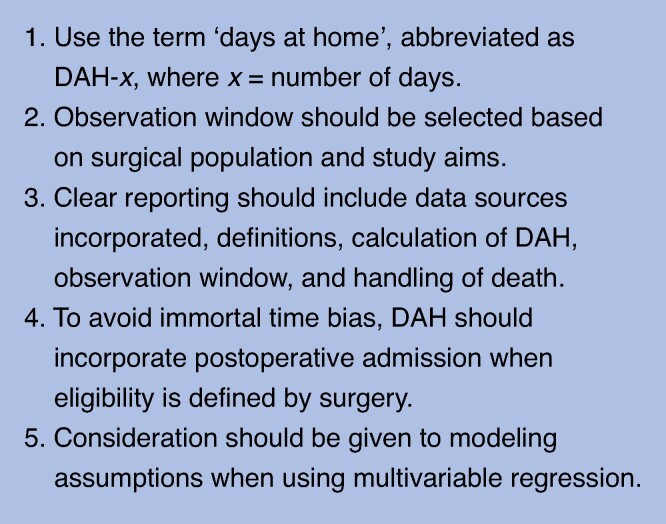
Recommendations for the use of ‘days at home’ in perioperative research

DAH appears to be a robust, valid, and accessible perioperative outcome that captures a holistic measure of the impact of surgery. By incorporating multiple measures of healthcare utilization, DAH is a comprehensive patient-centred metric, with a role in both research and policy. Globally, DAH is increasingly used across multiple surgical disciplines and study designs. It has been used in descriptive analyses, comparative effectiveness research, and clinical trials. However, there is significant heterogeneity in terminology, definitions, and analysis, as well as inconsistent reporting practices. These factors impact the interpretability and adoption of DAH as an outcome measure, while affecting the quality of the evidence base. Based on observations, recommendations for its standardization are provided to improve the use of DAH in perioperative settings (*Fig. 5*).

This review identified several strengths of DAH after surgery. First, DAH is comprehensive. By incorporating length of stay and healthcare utilization after discharge, DAH captures meaningful morbidity and functional impacts that alter length of stay and/or postoperative disposition. In addition, DAH captures recurrent events and, by incorporating mortality, avoids competing risk. Second, there is face validity in the use of DAH use as a patient-centred metric. Time at home has been found to be associated with both patient-reported outcomes, such as reduced depression and well-being, and functional outcomes^71,91–93^. In addition, prior work exploring values in the elderly and those at end of life found the majority of people value time spent at home^94^. Although it is acknowledged that time at home may not always reflect better quality of life, such as home environments with insufficient supports, DAH remains important to reflect the landscape of life after surgery. Third, DAH is flexible to being adapted to multiple study designs. DAH can be used with a range of data availability and capture different measures of healthcare utilization depending on aims. Fourth, DAH has utility from a health systems perspective. Prior work has demonstrated DAH is inversely related to cost at a national level, thus supporting its role as an important proxy for cost-effective quality of care^59^. Finally, DAH is a valid measure. There has been consistent demonstration of construct validity (DAH associated with patient-, surgery-, and system-level factors known to be related to outcomes after surgery) and criterion validity (DAH associated with other postoperative outcomes) across multiple healthcare delivery settings and patient populations, as outlined in seven validation studies included in this review^8,36,46,49,59,66,89^. Together, these factors point towards the robustness of DAH as an outcome and support its rigour as a patient-centred tool in research, policy, and quality-improvement initiatives.

The rationale for use of DAH after surgery was consistent across studies; however, there was variability in several aspects of its use. Terminology varied, with the number of unique terms reflecting its recent introduction into the perioperative literature. In addition to terminology, definitions of DAH varied. This is not surprising as the definition of DAH changes depending on which measures of healthcare utilization are incorporated. In resource-intensive studies, calculation of DAH incorporated not only days in healthcare institutions but also outpatient visits, commute time, and time receiving outpatient treatments^44,80^. Other studies used routinely collected registry or administrative data to calculate DAH. In addition to facilitating robust and feasible observational research, this approach creates the opportunity for policy initiatives using DAH, as has been pursued by the Medicare Payment Advisory Commission^95^. Accordingly, the accessibility of DAH favours a potential role for its use in pragmatic registry-linked trials. The duration of the observation window differed among studies, reflecting different research objectives and study populations. In populations with prolonged postoperative stays or substantial morbidity, an intermediate observation window ranging from 60 to 180 days may allow for more meaningful detection of differences. Regardless of the observation window, DAH should incorporate the hospital stay after surgery to avoid immortal time bias^96^. This was noted to be an issue with the operationalization of DAH in a few studies^24,30,65,84^. The variability in how DAH is defined may limit its widespread use, given challenges with interpretation and synthesis of data. However, consensus terminology would improve understanding of the construct captured by DAH and facilitate dissemination of a coordinated body of literature. The authors have chosen the term ‘days at home’ to represent this construct due to its frequency of use, generalizability, and simplicity. Although days alive and out of hospital was most frequently used, this term is not applicable to all studies as DAH can incorporate measures of healthcare utilization beyond hospitalizations.

A major consideration in the use of DAH is handling of mortality due to the implications of competing risk^97^. The most common method of handling death was to subtract dead days from the observation window, treating dead days as equivalent to institution days. In this case, two patients within a cohort may have equal DAH, with one having died in the observation window after being discharged after surgery. The second most common method was handling death as a DAH of zero. This would result in death appearing equivalent to all days spent in healthcare institutions within an observation window. Although these are not equivalent outcomes, this would reflect the weight of death as representing the worst outcome after surgery. Consideration should be given to the length of the observation window and the mortality rate of the study population when deciding on handling of mortality. Regardless of approach, clear reporting is needed and sensitivity analyses should be considered that use alternative methods of handling death to support study findings.

The heavily skewed distribution of DAH, with a preponderance of zeros, poses unique considerations when using multivariable regression. Quantile regression was the most common method used secondary to the lack of distributional assumptions required, and ability to regress on the median, resulting in a model robust to outliers typically present when using DAH^98,99^. In contrast, despite there likely being violations in model assumptions used in linear (ordinary least squares) regression (that is homoscedasticity of residuals, linearity), multiple studies used this method^100^. In addition, when handling DAH as a count, overdispersion should be tested before using the Poisson distribution as it is likely that negative binomial would be favoured^101^. Future work using multivariable analyses should explore the assumptions inherent in regression models to avoid biased estimates.

The interpretation of findings related to differences in DAH should move beyond statistical significance and consider clinical meaning. Prior work defined the minimal clinically importance difference in DAH 30 days after surgery to be 3 days via patient surveys^102^. This aligns with work exploring minimally important differences via analytic methods where a difference of 11 days when measuring DAH 90 days after surgery was meaningful^49^. Together, these results imply an approximate 10% change in DAH being relevant; however, some work has suggested a 1 day difference is important in DAH^59^. Overall, it may be argued that the true minimally important difference is relative, depending on the distribution and variability of DAH in a specific population. From a resource-utilization perspective, the cost implications of 1 day likely support this difference as meaningful^59^. As the use of DAH continues to increase, future work should continue to explore this concept to better define what is meaningful.

This review has limitations. First, in keeping with scoping review methodology, this review did not include quality or bias assessment; instead, it mapped the literature on this emerging measure to guide future use. In addition, the construct captured by DAH is identified by an evolving number of terms. Although the search was run iteratively to capture additional terms identified in the search, it is possible that studies were missed. However, the overall impact and observations made are unlikely to change. Lastly, although the included studies were not restricted with regard to geographical region, publications were restricted to English-language publications, limiting wider capture.

DAH after surgery is a feasible, robust, and patient-centred outcome measure. This review highlights its flexibility, with regard to applications to different study designs and patient populations, and provides recommendations for its future use (*Fig. 5*). As DAH after surgery continues to be increasingly used, there is a need to work towards coordinated terminology and improved reporting to ensure optimal use of this novel outcome as a patient-centred policy and research tool.

## Supplementary Material

znae278_Supplementary_Data

## Data Availability

Full data extracted from the included papers are provided in the Supplementary material.
