# Supplementary material for: Days at home after surgery as a perioperative outcome: scoping review and recommendations for use in health services research
Source: Br J Surg. 2024 Dec 4;111(12):znae278. doi: 10.1093/bjs/znae278 (PMC11630023; doi:10.1093/bjs/znae278)
Supplement: znae278_Supplementary_Data [file znae278_supplementary_data.zip › Supplementary_material.docx]

**Days at home after surgery as a perioperative outcome: Scoping review and recommendations for use in health services research**

Tiago Ribeiro, MD^1 2^; Armaan K Malhotra, MD ^1 2^; Adom Bondzi-Simpson, MD^1 2^; Antoine Eskander MD ScM ^2 6 7^; Negar Ahmadi MD MPH^1^; Frances C Wright MD MEd^1 2 7^; Daniel I McIsaac MD MPH^8 9 10^; Alyson Mahar, PhD^3^; Angela Jerath, MD MSc^2 4 5 6^; Natalie Coburn, MD MPH^1 2 6 7^; Julie Hallet, MD MSc^1 2 6 7^

1. Department of Surgery, University of Toronto, Toronto, Ontario, Canada
2. Institute of Health Policy Management and Evaluation, University of Toronto, Toronto, Ontario, Canada
3. School of Nursing, Queen’s University, Kingston, Ontario, Canada
4. Department of Anesthesiology and Pain Medicine, University of Toronto, Toronto, Ontario, Canada
5. Department of Anesthesia, Sunnybrook Health Sciences Centre, Toronto, Ontario, Canada
6. Clinical Evaluative Sciences, Sunnybrook Research Institute, Toronto, Ontario, Canada
7. Division of Surgical Oncology, Odette Cancer Centre - Sunnybrook Health Sciences Centre, Toronto, Ontario, Canada
8. Department of Anesthesiology & Pain Medicine, University of Ottawa, Ottawa, Ontario
9. School of Epidemiology & Public Health, University of Ottawa, Ottawa, Ontario,
10. The Ottawa Hospital Research Institute, Ottawa, Ontario, Canada.

**Corresponding author**:

Dr. Julie Hallet

2075 Bayview avenue, T2-102, Toronto, Ontario, Canada, M4N 3M5

T. 416-480-4774; F. 416-480-6002; E. Julie.hallet@sunnybrook.ca

**Supplementary Materials - Index**

| **Supplementary Figures and Tables** |  |
| --- | --- |
| Table S1. Medline search strategy | *pag. 2* |
| Table S2. Embase search strategy | *pag. 3* |
| Table S3. Scopus search strategy | *pag. 5* |
| Table S4. Summary data extraction table | *pag. 6* |
|  |  |

**Supplementary Figures and Tables**

**Table S1.** Medline search strategy

| **Search number** | **Terms and subject headings** | **Results** |
| --- | --- | --- |
| 1 | (Days at home or time at home or "days alive and out of hospital" or "days alive and out of the hospital" or "days alive out of hospital" or days alive out of the hospital or days alive at home or "days alive and at home" or home time or healthy days at home or time spent at home or "time spent alive and out of hospital" or "time spent alive and out of the hospital" or "time spent alive and at home" or time toxicity or opportunity cost* or institution day* or institution time or time in institution* or time spent away from home or time spent away from the home or "time alive and at home" or time alive at home or time at home).tw,kf. | 3062 |
| 2 | specialties, surgical/ or colorectal surgery/ or general surgery/ or gynecology/ or neurosurgery/ or obstetrics/ or ophthalmology/ or orthognathic surgery/ or orthopedics/ or otolaryngology/ or surgery, plastic/ or surgical oncology/ or thoracic surgery/ or traumatology/ or urology/ | 217838 |
| 3 | (surgery or surgeries or surgical or resection* or procedure* or incision* or excision* or operation*).tw,kf. | 3071349 |
| 4 | 2 or 3 | 3196976 |
| 5 | 1 and 4 | 441 |

**Table S2.** Embase search

| **Search number** | **Terms or subject headings** | **Results** |
| --- | --- | --- |
| 1 | (Days at home or time at home or "days alive and out of hospital" or "days alive and out of the hospital" or "days alive out of hospital" or days alive out of the hospital or days alive at home or "days alive and at home" or home time or healthy days at home or time spent at home or "time spent alive and out of hospital" or "time spent alive and out of the hospital" or "time spent alive and at home" or time toxicity or opportunity cost* or institution day* or institution time or time in institution* or time spent away from home or time spent away from the home or "time alive and at home" or time alive at home or time at home).tw,kf. | 4934 |
| 2 | exp laser surgery/ or exp off pump coronary surgery/ or exp transanal endoscopic surgery/ or exp gynecologic surgery/ or exp laparoendoscopic single site surgery/ or exp cerebrovascular surgery/ or exp enhanced recovery after surgery/ or exp endoscopic surgery/ or exp pelvis surgery/ or exp adrenal surgery/ or exp robot assisted surgery/ or exp heart surgery/ or exp anus surgery/ or exp biliary tract surgery/ or exp experimental surgery/ or exp ear surgery/ or exp vitreoretinal surgery/ or exp thyroid surgery/ or exp thymus surgery/ or exp tendon surgery/ or exp laparoscopic surgery/ or exp maxillofacial surgery/ or exp cytoreductive surgery/ or exp minor surgery/ or exp aneurysm surgery/ or exp coronary artery surgery/ or exp urethra surgery/ or exp endoscopic endonasal surgery/ or exp intestine surgery/ or exp refractive surgery/ or exp foot surgery/ or exp breast surgery/ or exp endocrine surgery/ or exp transoral robotic surgery/ or exp nerve surgery/ or exp rectum surgery/ or exp cardiovascular surgery/ or exp knee surgery/ or exp carotid artery surgery/ or exp ligament surgery/ or exp skull surgery/ or exp coronary artery bypass surgery/ or exp endoscopic sinus surgery/ or exp retina detachment surgery/ or exp open surgery monopolar electrosurgical electrode/ or exp endovascular surgery/ or exp "aortic root surgery"/ or exp endodontic surgery/ or exp oral surgery/ or exp stomach surgery/ or exp preprosthetic surgery/ or exp emergency surgery/ or exp failed back surgery syndrome/ or exp gastric bypass surgery/ or exp uterine tube surgery/ or exp spleen surgery/ or exp glaucoma surgery/ or exp retina surgery/ or exp nephron sparing surgery/ or exp colon surgery/ or exp pancreas surgery/ or exp microvascular surgery/ or exp mitral valve surgery/ or exp transsphenoidal surgery/ or exp abdominal surgery/ or exp general surgery/ or exp aortic arch surgery/ or exp nose surgery/ or exp uterus surgery/ or exp vein surgery/ or exp skin surgery/ or exp colorectal surgery/ or exp ascending aorta surgery/ or exp Mohs micrographic surgery/ or exp pediatric surgery/ or exp knee ligament surgery/ or exp brain surgery/ or exp lung surgery/ or exp craniofacial surgery/ or exp eye surgery/ or exp cancer surgery/ or exp natural orifice transluminal endoscopic surgery/ or exp "head and neck surgery"/ or exp male genital system surgery/ or exp bladder surgery/ or exp video assisted surgery/ or exp heart valve surgery/ or exp larynx surgery/ or exp conversion to open surgery/ or exp ear nose throat surgery/ or exp geriatric surgery/ or exp vagina surgery/ or exp coagulation surgery/ or exp corneal surgery/ or exp trachea surgery/ or exp robotic surgery simulator/ or exp urologic surgery/ or exp dental surgery/ or exp spine surgery/ or exp strabismus surgery/ or exp liver surgery/ or exp thoracic aortic surgery/ or exp minimally invasive cardiac surgery/ or exp surgery/ or exp open surgery/ or exp descending aortic surgery/ or exp artery surgery/ or exp orthopedic surgery/ or exp decompression surgery/ or exp aortic surgery/ or exp endoscopic pituitary surgery/ or exp plastic surgery/ or exp esthetic surgery/ or exp video assisted thoracoscopic surgery/ or exp gastrointestinal surgery/ or exp facial nerve surgery/ or exp ambulatory surgery/ or exp prostate surgery/ or exp kidney surgery/ or exp spinal cord surgery/ or exp robotic knee surgery system/ or exp joint surgery/ or exp cardiac surgery intensive care unit/ or exp off pump surgery/ or exp hand surgery/ or exp meniscal surgery/ or exp hip surgery/ or exp orthognathic surgery/ or exp laser refractive surgery/ or exp pituitary surgery/ or exp plastic surgery implant/ or exp ureter surgery/ or exp bypass surgery/ or exp throat surgery/ or exp orthodontic surgery/ or exp ultrasound surgery/ or exp vascular surgery/ or exp middle ear surgery/ or exp second look surgery/ or exp face surgery/ or exp urinary tract surgery/ or exp reconstructive surgery/ or exp elective surgery/ or exp computer assisted surgery/ or exp breast-conserving surgery/ or exp shoulder surgery/ or exp arthroscopic surgery/ or exp minimally invasive surgery/ or exp ankle surgery/ or exp computer assisted surgery system/ or exp thorax surgery/ or exp esophagus surgery/ or exp stapes surgery/ or exp open heart surgery/ or exp bariatric surgery/ or exp major surgery/ or exp wrist surgery/ or exp robotic hip surgery system/ | 6242449 |
| 3 | (surgery or surgeries or surgical or resection* or procedure* or incision* or excision* or operation*).tw,kf. | 5102967 |
| 4 | 2 or 3 | 8323665 |
| 5 | 1 and 4 | 1032 |

**Table S3.** Scopus search

| **Search Number** | **Terms** | **Results** |
| --- | --- | --- |
| 1 | (TITLE-ABS-KEY ("days at home" OR "time at home" OR "days alive and out of hospital" OR "days alive and out of the hospital" OR "days alive out of hospital" OR "days alive out of the hospital" OR"days alive at home" OR "days alive and at home" OR "home time" OR "healthy days at home" OR "time spent at home" OR "time spent alive and out of hospital" OR "time spent alive and out of the hospital" OR "time spent alive and at home" OR "time toxicity" OR "opportunity cost*" OR "institution days"OR "institution time" OR "time in institution*" OR “ time spent away from home” OR “time spent away from the home” OR "time alive and at home" OR “time alive at home” )) | 12727 |
| 2 | (TITLE-ABS-KEY("surgery" OR "surgeries" OR "surgical" OR "resection*" OR "procedure*" OR "incision*" OR "excision*" OR "operation*")) | 10347887 |
| 3 | 1 and 2 | 641 |

**Table S4.** Summary data extraction table

| **Author (year)** | **Study country** | **Study design** | **Study type** | **Study aim** | **Patient Population** | **Exposure or comparators** | **Outcome term: Definition** | **Main findings** |
| --- | --- | --- | --- | --- | --- | --- | --- | --- |
| Alexander *et al.* (2022) | New Zealand | Retrospective cohort study | Descriptive | Evaluate the association between complications and DAH | Adults undergoing acute or elective laparoscopic cholecystectomy | Complications and conversion to open versus none | **Days alive and out of hospital**: Incorporated inpatient days, other healthcare facility and dead days. Measured at 90 days. | Complications after surgery were associated with worse DAH |
| Alkadri *et al.* (2022) | Canada | Retrospective cohort study | Descriptive | Estimate association of frailty with DAH | Adults requiring emergency general surgery and ICU admission | Frailty | **Days at home**: Incorporated inpatient days, long-term care days, and dead days. Measured at 30 days. | Frailty was associated with worse DAH |
| Arya *et al.* (2022) | United States of America | Prospective cohort study | Descriptive | Examine association between post-op DAH and quality of life, functional status and regret | United states veterans age ≥65 years who underwent inpatient surgery | Quality of life, functional status, and decisional regret | **Home time:** Incorporated any days in health care institutions. Measured as a proportion of time from surgery to survey date spent at home. | Higher DAH was associated with better quality of life and functional status after surgery |
| Awada *et al.* (2022) | Denmark | Retrospective cohort study | Descriptive | Evaluate days alive and out of hospital following surgery for oral squamous cell carcinoma | Adults undergoing surgery for oral squamous cell carcinoma | N/A | **Days Alive and Out of Hospital**: Incorporated inpatient stay, readmissions days, and days dead. Measured at 30 and 365 days. | Median DAH-30 after surgery was 25 days and 356 days for DAH-365 |
| Barendse *et al.* (2018) | Netherlands | Clinical trial | Effectiveness | Compare outcomes and cost-effectiveness of endoscopic mucosal resection with transanal endoscopic microsurgery for large rectal adenoma | Adults with large rectal adenomas ≥3cm without malignant features | Endoscopic mucosal resection vs. transanal endoscopic microsurgery | **Days alive and out of hospital** and recurrence free: Definition unclear. | No difference in DAH for endoscopic mucosal resection and transanal endoscopic microsurgery |
| Barry *et al.* (2022) | Australia | Quasi-experimental design | Effectiveness | Assess the impact of closed-incision negative pressure wound therapy | Elective or emergent vascular surgery with groin incision | Closed-incision negative pressure wound therapy vs. none | **Days alive and out of hospital**: Incorporated inpatient hospital days. Measured over 90 days. | Closed-incision negative pressure wound therapy was associated with increased DAH |
| Behman *et al.* (2023) | Canada | Retrospective cohort study | Effectiveness | Compare long-term healthcare dependency, between older adults undergoing minimally invasive surgery and those undergoing open resection for colorectal cancer | Older adults ≥70 years undergoing surgery for colorectal cancer | Minimally invasive surgery vs. open | **Time at home**: Incorporated day in acute inpatient care, emergency department, mental health institutions, rehabilitation, or long-term care. Dichotomized into low (≤14 days/year) and high (>14 days/year). | Minimally invasive surgery for colorectal cancer resection was associated with higher probability of high home time in the 5 years after surgery |
| Bell *et al.* (2019) | Sweden | Retrospective cohort study | Validation | Demonstrate criterion and predictive validity of DAH30 using Swedish national data | Adults ≥17 years who underwent elective or non-elective surgery. Excluding nursing home patients | N/A | **Days at home:** Incorporated inpatient hospital. Measured at 30 days. Mortality DAH30=0. | Demonstrated DAH is valid, has content validity, and predictive validity for one-year survival. DAH had stronger prognostic utility than length of stay |
| Bennett *et al.* (2022) | Canada | Retrospective cohort study | Descriptive | Describe short- and long-term utilization of homecare services, and time at home, for older adults undergoing hepatopancreatobiliary cancer surgery | Older adults aged ≥70 years, undergoing either hepatectomy or pancreatectomy, for hepatopancreatobiliary cancer | N/A | **Time at home**: Incorporated day in acute inpatient care, emergency department, mental health institutions, rehabilitation, or long-term care. Dichotomized into low (≤14 days/year) and high (>14 days/year). | Probability of high DAH was 40% at 1 year and 28.1% at 5 years. |
| Bergengren *et al.* (2023) | Sweden | Retrospective cohort study | Effectiveness | Investigate whether robot assisted radical cystectomy led to improvements in outcomes for patients undergoing surgery for bladder cancer | Robot assisted radical cystectomy vs. open radical cystectomy for bladder cancer | Robotic vs. open radical cystectomy | **Days alive and out of hospital:** Measured over 90 days. Definition unclear. | Robotic approach was associated with 3.7 day increased DAH-90 |
| Bittermann *et al.* (2018) | United States of America | Retrospective cohort study | Descriptive | Describe post liver transplant health care utilization and evaluate predictors of DAH | Adults undergoing liver transplantation | N/A | **Days alive and out of hospital**: Incorporated inpatient hospital days. Measured at 1 year. | Pre-liver transplant hospitalization was associated with worse DAH in 1 year |
| Bolshinsky *et al*. (2022) | Australia | Retrospective cohort study | Descriptive | Explore the predictive value of several prognostic factors including cardiopulmonary exercise testing for adults undergoing colorectal cancer surgery | Adults undergoing colorectal cancer surgery and cardiopulmonary exercise testing | N/A | **Days at home:** Measured over 90 days. Definition unclear. | Low hemoglobin and albumin were associated with reduced DAH-90 in univariable analysis |
| Brennan *et al*. (2017) | United States of America | Retrospective cohort study | Effectiveness | Determine safety and effectiveness of transcatheter aortic valve replacement vs surgical, in intermediate- and high-risk patients | Adults 65-90 years undergoing surgery for severe, symptomatic aortic valvular insufficiency at intermediate- or high-risk | transcatheter aortic valve replacement vs surgical | **Days alive and out of hospital**. Incorporated inpatient hospital days and measured after discharge. Measured at 1 year. | DAH similar between transcatheter aortic valve replacement and surgical aortic valve replacement |
| Campfort *et al.* (2022) | France | Prospective cohort study | Descriptive | Evaluate association between post-operative recovery and 1-month complications | Adults undergoing elective surgery. | N/A | **Days alive and out of hospital**: Definition unclear, calculated via medical record and phone call. Measured at 30 days. | Poor quality of recovery score was associated with worse DAH-30 |
| Catto *et al*. (2022) | United Kingdom | Clinical trial | Effectiveness | Compare total intracorporal robot-assisted radical cystectomy with open radical cystectomy among patients with non-metastatic bladder cancer | Adults with non-metastatic bladder cancer undergoing radical cystectomy | Total intracorporal robotic radical cystectomy | **Days alive and out of hospital**: Incorporated inpatient stay, readmissions, and death. Measured at 90 days. | Robotic approach led to 2.2 day increase in DAH-90 |
| Chesney *et al.* (2020) | Canada | Retrospective cohort study | Descriptive | Examine time at home for older adults in the 5 years after cancer resection | Older adults aged ≥70 years who underwent surgery for a new diagnosis of solid malignant neoplasm | N/A | **Time at home**: Incorporated day in acute inpatient care, emergency department, mental health institutions, rehabilitation, or long-term care. Dichotomized into low (≤14 days/year) and high (>14 days/year). | Probability of high DAH was 70%, 61%, and 53% at year 1, 3 and 5, respectively. Female sex and recent diagnosis were associated with superior probability of high DAH. Advancing age and worsening material deprivation associated with inferior probability of high DAH. |
| Chung *et al.* (2023) | United States of America | Secondary analysis of clinical trial | Descriptive | Describe postprocedural course of high-risk patients undergoing aortic valve replacement | High risk adult patients undergoing aortic valve replacement enrolled in a trial | Surgical vs. transcatheter aortic valve replacement | **Days at home:** Incorporated emergency department visit, inpatient days, rehabilitation, long-term care, acute care, and skilled nursing facility. Measured at 30, 90, 365 and 1825 days. | High-risk patients undergoing transcatheter aortic valve replacement spent on average 27 more DAH in first year |
| Chung *et al.* (2022) | United States of America | Secondary analysis of clinical trial | Descriptive | Describe DAH-365 of intermediate-risk patients undergoing aortic valve replacement | Intermediate risk adults undergoing aortic valve replacement | Surgical vs. transcatheter aortic valve replacement | **Days at home:** Incorporated emergency department visit, inpatient days, rehabilitation, long-term care, acute care and skilled nursing facility. Measured at 30, 90, and 365 days. | Intermediate-risk patients undergoing transcatheter aortic valve replacement had 15 more DAH-365 |
| Frasco *et al.* (2023) | United States of America | Retrospective cohort study | Descriptive | Identify differences of DAH-365 after deceased liver transplant by graft types | Adults undergoing primary deceased donor orthotopic liver transplant | Donor after cardiac death vs. donor after brain death | **Days alive and out of hospital**: Incorporated days in hospital or rehab facility. Mortality DAH=0 if original admission, if not than days dead = institution days. Measured at 365 days. | Graft type was not associated with decreased DAH. Age, race, transfusion, and cold ischemia time were associated with DAH. |
| Fung *et al.* 2022) | China | Clinical trial | Effectiveness | Examine the effect and feasibility of using preoperative iron isomaltoside for treating iron deficiency | Adults with colorectal cancer scheduled for curative surgery and iron deficiency anemia | Intravenous iron isomaltoside pre-operative | **Days at home**: Incorporated inpatient stay, discharge deposition, readmission and postoperative mortality. Measured at 30 days. | No difference in DAH-30 between groups. |
| Gaber *et al.* (2022) | United States of America | Retrospective cohort study | Effectiveness | Assess the comparative effectiveness of trimodality therapy vs definitive chemoradiation in a population of older US adults with esophageal cancer | Older adults aged 66-79 years with newly diagnosed locally advanced esophageal cancer undergoing trimodality (chemo, rads, surgery) vs. definitive chemoradiation | Trimodality therapy vs. definitive chemoradiation | **Days at home:** Measured at 5 years. Definition unclear. | Trimodality therapy was associated with more DAH |
| Gilhooly *et al.* (2018) | United Kingdom | Prospective cohort study | Descriptive | Evaluate two previously developed post-op risk prediction tools for bariatric surgery and evaluate independent predictors of poor weight loss. DAH used to capture life impact | Adults undergoing bariatric surgery | N/A | **Days alive and out of hospital:** Definition unclear. Measured at 30 days. Dichotomized at 28 days. | Low DAH was predictive of poor weight loss |
| Grieve *et al*. (2023) | United Kingdom | Retrospective cohort study | Effectiveness | Evaluate clinical effectiveness and cost-effectiveness of emergency surgery | Adults undergoing emergency general surgery | Surgery vs. non-surgical treatment strategies | **Days alive and out of hospital**: Incorporated inpatient hospitalization data. Measured at 90 days. | DAH-90 similar for surgical and non-surgical strategies |
| Grudzinski *et al*. (2022) | Canada | Retrospective cohort study | Descriptive | Compare predictive accuracy of frailty instruments in adults undergoing emergency general surgery. DAH was secondary outcome | Adults ≥65 undergoing emergency general surgery | Frailty tools | **Days alive at home:** Incorporated acute care hospital admissions and other institutions. Measured at 30 and 365 days. | Frailty instruments improved prediction of DAH |
| Grudzinski et al. (2022) | Canada | Retrospective cohort study | Descriptive | Determine predictive accuracy of frailty instruments in predicting postoperative outcomes in older adults | Adults ≥65 undergoing elective non-cardiac surgery | Frailty tools | **Days alive at home:**  Incorporated inpatient acute care and other health care institutions. Measured at 30 and 365 days. | Frailty instruments improved prediction of DAH |
| Guttman *et al*. (2021) | Canada | Retrospective cohort study | descriptive | Evaluate association between emergency general surgery admission and probability of OA being alive and at home 5 years later | Older adults age ≥65 undergoing emergency general surgery | N/A | **Time spent alive and at home**: Sum of days until death or admission to nursing home after discharge from surgery. |  |
| Hallet et al. (2022) | Canada | Retrospective cohort study | Effectiveness | Compare long-term healthcare dependency outcomes of older adults undergoing video-assisted thoracoscopic surgery and those undergoing open resection for lung cancer | Older adults (≥70 years) who underwent surgical resection for lung cancer | Open vs. video-assisted thoracoscopic lung resection | **Time at home**: Incorporated day in acute inpatient care, emergency department, mental health institutions, rehabilitation, or long-term care. Dichotomized into low (≤14 days/year) and high (>14 days/year). | Video-assisted thoracoscopic surgery was associated with higher probability of high time-at-home |
| Hallet *et al*. (2022) | Canada | Retrospective cohort study | Descriptive | Examine the association between preoperative frailty and remaining alive and at home in 5 years after cancer surgery in older adults | Older adults (≥70 years) undergoing surgery for new diagnosis of any cancer | Pre-operative frailty | **Time spent alive and at home**: Sum of days until death or admission to nursing home after discharge from cancer surgery. | Probability of being alive and at home after cancer surgery was lower for patient with preoperative frailty |
| Hirpara *et al*. (2022) | Canada | Retrospective cohort study | Effectiveness | Compare the long-term health dependency outcomes of older adults undergoing stereotactic body radiation therapy and surgery for Stage I Non-Small Cell Lung Cancer | Older adults (>70 years) with a new diagnosis of stage I Non-small cell lung cancer undergoing surgery or radiotherapy | Surgery versus stereotactic body radiation therapy | **(1) Days at home:** Incorporated days spent in hospital and other institutions. Measured at 12 months. **(2) Time spent alive and at home**: Time to death or admission to a nursing home from time of treatment to end of follow-up | Radiotherapy patients had a higher median DAH at 12 months and a lower probability of being alive and at home over 5 years following treatment |
| Huang *et al*. (2022) | Denmark | Retrospective cohort study | Descriptive | Evaluate days alive and out of hospital within 365 days after video-assisted thoracoscopic lobectomy within an enhanced recovery after surgery program | Adults with non-small cell lung cancer undergoing video-assisted thoracoscopic surgery in an enhanced recovery after surgery | N/A | **Days alive and out of hospital:** Incorporated inpatient acute care days and death. Measured at 30, 90, 180 and 365 days. | DAH in an enhanced recovery after surgery program reduced median DAH by 6 days |
| Hung *et al.* (2015) | United Kingdom | Prospective cohort study | Descriptive | Determine relative frequency of different causes of anemia and examine associations with anemia and outcomes in cardiac surgery | Adults with anemia undergoing cardiac surgery | N/A | **Days alive and out of hospital:** Definition unclear. Measured at 30 days. | Anemia was not associated with worse DAH |
| Hutchings *et al*. (2022) | United Kingdom | Retrospective cohort study | Descriptive | Assess effectiveness of emergency surgery vs. non-emergency surgery for emergency general surgery presentations | Adults presenting with one of 5 emergency general surgery presentations | Emergency surgery vs. other management | **Days alive and out of hospital:** Incoporated acute care hospitalization data. Measured at 90 days. Mortality = 0 | Similar DAH-90 following emergency general surgery and other strategies |
| Jerath *et al*. (2020) | Canada | Retrospective cohort study | Descriptive | Determine adjusted association of socio-economic status with DAH | Adults ≥40 undergoing elective high- and intermediate-risk noncardiac surgical procedures | Socio-economic status | **Days alive and out of hospital**: Incorporated hospitalizations. Mortality DAH=0. Measured at 30 days. | DAH increases with increased household affluence |
| Jerath et al. (2020) | Canada | Retrospective cohort study | Descriptive | Evaluate whether higher post-operative ICU rates was associated with improved DAH after elective major noncardiac surgical procedures | Adults ≥40 undergoing elective major noncardiac surgical procedures | Hospital specific ICU admission rate/ surgical procedure | **Days alive and out of hospital:** Incorporated hospitalizations. Measured at 30 days. Mortality DAH=0 | Higher ICU utilization did not increase DAH |
| Jerath et al. (2019) | Canada | Retrospective cohort study | Validation | Characterize DAH in surgical patients, describe construct validity, assess criterion validity | Adults ≥40 undergoing elective high- and intermediate-risk noncardiac surgical procedures | N/A | **Days alive and out of hospital:** Incorporated hospitalizations. Measured at 30 days. Mortality DAH=0 | DAH demonstrated construct and criterion validity. |
| Jorgensen *et al*. (2019) | Denmark | Retrospective cohort study | Descriptive | Evaluate DAH in a cohort of fast-track total knee arthroplasty and total hip arthroplasty patients | Adults undergoing unilateral or bilateral total hip or knee arthroplasty in a fast-track centre | N/A | **Days alive and out of hospital**: Incorporated hospitalizations, death and readmission if related to surgery. Measured at 30 and 90 days. | DAH is lower in high-risk patients |
| Kim *et al*. (2022) | South Korea | Retrospective cohort study | Descriptive | Investigate relationship between DAH-90 in non-anaemic iron-deficient patients undergoing valvular heart surgery | Adults undergoing elective valvular heart surgery with non-anaemic iron-deficiency | Non-anaemic iron-deficient versus non-anaemic non-iron deficiency | **Days alive and out of hospital**: Definition unclear. Measured at 90 days. | Median DAH-90 was 1 day shorter in iron-deficient group |
| Klein *et al*. (2020) | United Kingdom | Prospective cohort study | Descriptive | Assess the introduction and efficacy of preoperative IV iron pathway to treat anemia in patients before cardiac surgery | Adults awaiting cardiac surgery with anaemia | Anaemic patients treated with IV iron versus not | **Days alive and at home**: Incorporated all hospitalizations. Measured at 30 days. | No difference in outcomes of patients treated with IV iron versus untreated |
| Kunkel *et al*. (2021) | United States of America | Prospective cohort study | Descriptive | Evaluate whether delirium is independently associated with reduced DAH-90 | Adults >65 undergoing major non-intracranial surgery | N/A | **Days alive and at home:** Definition unclear. Measured at 90 days. | Non-delirious subjects had a greater DAH90 |
| Larsen *et al.* (2021) | Denmark | Retrospective cohort study | Descriptive | Describing morbidity and mortality burden associated with trans oral robotic surgery for cancer using days alive and out of hospital | Adults undergoing surgery for cancer | N/A | **Days alive and out of hospital:** Incorporated inpatient days and death. Measured at 365 days. | Median DAOH-365 was 357. |
| Lee *et al.* (2020) | United States of America | Retrospective cohort study | Descriptive | Examine impact of frailty on 1 year outcomes | Adults ≥65 years undergoing emergency general surgery procedures | Frailty | **Days at home**: Incorporated hospitalization days and death. Measured at 1 year. | Frail patients had fewer DAH |
| Lee et al. (2020) | United States of America | Retrospective cohort study | Descriptive | Examine outcomes after emergency general surgery vs. acute medical conditions at 1 year | Adults ≥65 years with urgent hospitalization for emergency general surgery procedure or acute medical diagnosis | Emergency general surgery vs acute medical conditions | **Days at home**: Incorporated hospitalization days and death. Measured at 1 year. | Emergency general surgery group experienced more DAH. |
| Lim *et al*. (2022) | United States of America | Retrospective cohort study | Descriptive | Determine time spent in multimodality care for patients with locoregional pancreatic ductal adenocarcinoma | Patients undergoing curative-intent resection for pancreatic ductal adenocarcinoma | N/A | **Total care time**: Incorporated preoperative, surgical, radiation and systemic therapy. For surgery included index hospital admission, f/u visits, ED visits, and other admissions. Other components included commute, time receiving rx etc. Also presented as percent of total survival time (TST). | Percentage of time spent in receipt of care was low. |
| M'Pembele *et al*. (2023) | Germany | Retrospective cohort study | Descriptive | Evaluate whether high-sensitivity troponin-I is suitable marker for risk stratification and prognosis after heart transplant | Adults undergoing heart transplant | N/A | **Days alive and out of hospital**: Incorporated hospitalization and death. Measured at 1 year. | Higher high-sensitivity troponin-I levels were associated with lower DAH |
| M'Pembele et al. (2022) | Germany | Retrospective cohort study | Descriptive | Investigate DAH in patients undergoing VA-ECMO for primary graft dysfunction after heart transplant | Adults undergoing heart transplant at one centre | N/A | **Days alive and out of hospital**: Incorporated hospitalization and death. Measured at 1 year. | Median DAH at 1 year was 293, DAH was high in non ECMO patients |
| M'Pembele et al. (2022) | Germany | Retrospective cohort study | Validation | Identify prognostic variables for DAH at 1yr after heart transplant | Adults undergoing heart transplant at one centre | N/A | **Days alive and out of hospital**: Incorporated hospitalization and death. Measured at 1 year. | Recipient diabetes, postoperative renal replacement therapy were independently associated with reduced DAH |
| Maibom *et al.* (2021) | Denmark | Retrospective cohort study | descriptive | To report short-term morbidity, length of stay, DAH within 90 days after radical cystectomy ( | Adults undergoing surgery for bladder cancer | N/A | **Days alive and out of hospital**: Incorporated inpatient admission, readmission, and death. Measured at 90 days, dichotomized at 80. | Median DAOH-90 was 80 |
| McGillion *et al*. (2021) | Canada | Clinical trial | Effectiveness | Determine whether virtual care with remote automated monitoring (increased DAH 31 days after non-elective surgery in adults | Adults undergoing non-elective surgery | Virtual care with remote automated monitoring vs. standard of care | **Days alive at home**: Definition unclear. Measured at 31 days. | No difference in DAH between groups |
| McIsaac *et al*. (2021) | Canada | Retrospective cohort study | Validation | Validate DAH as outcome in patients with hip fracture and estimate minimally important difference | Adults aged ≥50 with hip fracture admission | N/A | **Days alive at home**: Incorporated acute care, rehabilitation, long-term care, and respite care. Measured at 90 and 365 days. Mortality, DAH=0. | Predictive validity and construct validity of DAH were demonstrated. |
| McIsaac et al. (2021) | Canada | Retrospective cohort study | Descriptive | Estimate association of frailty with DAH after major cardiac surgery | Adults age ≥65 having one of 5 major cardiac surgeries | Frailty | **Days alive at home**: Incorporated acute care, rehabilitation, long-term care, and respite care. Measured at 30 and 365 days. Mortality, DAH=0. | Preoperative frailty was strongly associated with reduced DAH-30 and 365 |
| Mentias *et al.* (2022) | United States of America | Retrospective cohort study | descriptive | Evaluate association of 90-day risk-standardized home-time with other measures of performance and assess temporal stability. | Adults age ≥65 undergoing surgical aortic valve replacement, mitral valve replacement or coronary artery bypass grafting, or combination | N/A | **Risk standardized home time**: Incorporated days outside of any health care institutions and death. Measured at 90 days. | DAH-90 associated with annual surgical volume, readmission, mortality, length of stay. Findings were also stable across 7 years in a hospital. |
| Miles *et al*. (2018) | Australia | Retrospective cohort study | Descriptive | Compare iron-deficient group of patients undergoing cardiac surgery with iron replete | Patients undergoing elective CABG or single valve replacement | Iron-deficient vs. iron replete | **Days alive and out of hospital:** Definition unclear. Measured at 90 days. | Iron deficiency was associated with fewer DAH-90 |
| Miles *et al*. (2019) | Australia | Retrospective cohort study | Descriptive | Explore association between non-anaemic iron deficiency and day alive and out of hospital | Patients undergoing elective surgery for colorectal cancer | Non-anaemic and iron replete vs. non-anaemic and iron deficient | **Days alive and out of hospital:** Definition unclear. Measured at 90 days. | Iron deficient patients had 1.24 median reduced DAOH-90 |
| Miles *et al.* (2020) | Australia | Retrospective cohort study | Descriptive | Explore outcomes in women after abdominal in surgery based on hemoglobin levels | Women undergoing elective abdominal surgery with pre-operative hemoglobin measurement | Anaemic, borderline anaemic, vs. non-anaemic | **Days alive and out of hospital:** Definition unclear. Measured at 90 days. | Anaemic patients had lower DAH-30 and 90 |
| Miles *et al*. (2022) | Australia | Prospective cohort study | Descriptive | Evaluate whether iron-deficient patients without anemia have worse post-operative outcomes after elective cardiac surgery | Adults undergoing elective cardiac surgery | iron deficient vs. iron replete | **Days alive and at home:** Incorporated initial hospitalization, rehabilitation, subacute care and readmissions. Measured at 30 and 90 days. Mortality = 0. | No difference in DAH-30 or DAH-90 based on iron deficiency |
| Moore *et al*. (2022) | New Zealand | Retrospective cohort study | Effectiveness | Evaluate the effect surgical safety checklist has on outcomes | Adults with elective surgery | Surgical safety checklist vs. not | **Days alive and out of hospital**: Incorporated any day in hospital and death. Measured at 90 days. | Adults had more DAH-90 after surgical checklist implementation |
| Myles *et al.*(2017) | Australia | Secondary analysis of clinical trial | Validation | Evaluate days at home up to 30 days after surgery as a patient-centred metric in clinical trials and QI activities | Adults undergoing surgery | N/A | **Days at home**: Incorporated hospitalization data. Measured at 30 days. Mortality = 0. | DAH30 has construct validity |
| Noly *et al*. (2023) | United States of America | Retrospective cohort study | Descriptive | Compare outcomes pre- and post left ventricular assist device insertion. | Medicare beneficiaries (≥19) undergoing left ventricular assist device insertion |  | Percentage of **Days alive and out of hospital**: Incorporated hospitalization, rehabilitation, hospice, nursing days and death. Measured at 180 days (pre) and 365 days (post). | Pre-left ventricular assist device implantation DAH associated with post-insertion DAH. |
| Oh *et al*. (2023) | South Korea | Retrospective cohort study | Descriptive | Evaluate the association of DAH at 30 and 60 days with mortality and composite cardiac events after off pump CABG | Adults undergoing off pump CABG |  | **Days alive and out of hospital**: Incorporated hospitalization, excluded deaths. Measured at 30 and 60 days. | Short DAH predicted long term mortality and adverse cardiac events. |
| Pavol *et al*. (2021) | United States of America | Retrospective cohort study | Descriptive | Examine the relationship between cognition and DAH after left ventricular assist device implantation | Adults with advanced heart failure who underwent left ventricular assist device implantation | N/A | **Days alive and out of hospital**: Definition unclear. Measured until death, heart transplant or 900 days. Reported as a percentage. | Cognition predicted DAH |
| Plenge *et al.* (2020) | South Africa | Prospective cohort study | Descriptive | Report the quality of postoperative recovery after enhanced recovery protocols in South Africa for patients undergoing total hip or knee arthroplasty | Adults undergoing total hip or knee arthroplasty |  | **Days alive and at home** Determined through patient interviews. Incorporated acute care, re-admission, discharge disposition. Measured at 30 days. | No difference in DAH between tertiary and non-tertiary units. |
| Reilly *et al.* (2022) | Australia | Retrospective cohort study | Validation | Evaluated association between DAH and patient, surgical factors, and hospital cost | Adults undergoing any surgery | N/A | **Days alive and at home**: Incorporated inpatient hospitalization, nursing home, re-admission. Measured at 30 days. Mortality = 0. | DAH declined with age, comorbidity, ASA, physical status, surgical severity, emergency admissions, longer duration, women. Increase DAH also associated with lower cost |
| Roth *et al*. (2022) | Germany | Retrospective cohort study | Descriptive | Identify perioperative factors that impact DAH 1 year after surgery | Adults who received left ventricular assist device due to ischaemic heart disease, or dilated cardiomyopathy |  | **Days alive and out of hospital**: Incorporated hospitalization and death. Measured at 1 year. | Increased DAH associated with comorbidities, worse cardiac function |
| Roth *et al*. (2022) | Germany | Retrospective cohort study | Descriptive | Evaluate the impact of donor CPR on DAH in patients undergoing heart transplant | Adult heart transplant | CPR | **Days alive and out of hospital**: Incorporated hospitalization and death. Measured at 1 year. | No significant difference in DAH for CPR patients |
| Schick *et al*. (2021) | Germany | Prospective cohort study | Descriptive | Investigate the impact of endothelial dysfunction on the Days at Home within 30 days after surgery | Adults undergoing major abdominal surgery | Flow mediated dilation | **Days at Home**: Definition unclear. Measured at 30 days. | Flow mediated dilation was not a predictor of DAH-30 |
| Scott *et al*. (2021) | Denmark | Prospective cohort study | Effectiveness | Investigate DAH for patients with oropharyngeal squamous cell carcinoma treated with either transoral robotic surgery or radiotherapy | Patients with oropharyngeal squamous cell carcinoma eligible for surgery or radiotherapy | Transoral robotic surgery vs. radiotherapy | **Days alive and out of hospital:** Incorporated inpatient days for 30 days. At 180 days incorporated outpatient appointments and treatments. Measured at 30 and 180 days. | DAH was higher in patients treated with transoral robotic surgery compared to radiotherapy |
| Shah *et al.* (2022) | United States of America | Retrospective cohort study | Effectiveness | Determine effectiveness of CABG compared to percutaneous coronary intervention on DAH compared to traditional major adverse cardiac event outcomes | Adults undergoing revascularization procedure | CABG vs. PCI | **Days alive and out of hospital**: Incorporated hospitalization, long-term care, rehabilitation, skilled nursing, excluding index hospitalization. Measured at 1, 3 and 5 years. | CABG associated with greater number of DAH compared to PCI that increased over time |
| Shah et al. (2020) | United States of America | Retrospective cohort study | Descriptive | Evaluate association of dementia with traditional and patient centred outcomes in patients undergoing vascular procedures | Adults undergoing inpatient vascular procedures | Dementia | **Home time:** Incorporated admission and measured as percentage of days home to date of censoring or completion of calendar year | Patients with dementia spent a lower fraction of time at home after discharge |
| Shaw *et al.* (2022) | Canada | Retrospective cohort study | Descriptive | Assess impact increased frailty has on post-operative survival and outcomes | Adults undergoing any cancer surgery | N/A | **Days at home:** Incorporated acute inpatient care, emergency department, rehabilitation facilities or long-term care. Measured at 30 and 365 days. | Increased frailty resulted in fewer DAH. |
| Sokas *et al.* (2021) | United States of America | Retrospective cohort study | Descriptive | Examine association of frailty with intensity of end-of-life care for older adults who undergo emergency general surgery and die within one year | Medicare beneficiaries ≥66 year who underwent one of five emergency general surgery procedures and die within 1 year | Frailty | **Days at home:** Incorporated inpatient hospitalization, nursing. Measured after discharge from surgery. | Frailty resulted in fewer days at home |
| Soukkio *et al.* (2021) | Finland | Clinical trial | Effectiveness | Evaluate effect physical exercise program on DAH, health care use, function in patients with hip fractures | Adults age ≥60 years with operated hip fracture living at home | Supervised physical exercise 2x/week | **Days lived at home**: Incorporated hospitalization, nursing home, long term care and death. Measured at 24 months. | No significant difference in DAH |
| Spurling *et al.* (2022) | United Kingdom | Retrospective cohort study | Validation | Assess construct and predictive validity in DAH after emergency laparotomy | Adults undergoing emergency laparotomy |  | **Days alive and out of hospital**: Incorporated hospitalization. Measured at 30 days. Mortality =0. | Construct and predictive validity for DAH in emergency laparotomy |
| Suskind *et al.* (2020) | United States of America | Retrospective cohort study | Descriptive | Describe time spent away from home after high-risk cancer surgery | Medicare beneficiaries who are nursing home residents undergoing high risk surgery for any cancer | N/A | **Time spent at home:** Incorporated inpatient hospitalizations and nursing home. Measured at 1 year. Reported as proportion. | Adults who died within 1 year had a lower percentage of home time. |
| Tenge *et al.* (2022) | Germany | Retrospective cohort study | Effectiveness | Compare DAH in hemodynamically stable patients awaiting heart transplant on regular wait list vs patient undergoing left ventricular assist device implant | Adults undergoing left ventricular assist device | Left ventricular assist device implantation versus not | **Days alive and out of hospital:** Incorporated hospitalization and mortality. Measured at 365 days. | Median DAH at 1yr was no different between groups awaiting heart transplant |
| Tillmann *et al*. (2021) | Canada | Retrospective cohort study | Descriptive | Evaluate the relationship between unexpected ICU admission and long-term functional outcomes of older adults after high-intensity cancer surgery | Older adults (≥70 years) who underwent high risk surgery for any cancer | Unexpected post-operative ICU admission | **Time alive and at home**: Measured by time to nursing home admission or death. Followed for 5 years. | Older adults with unexpected ICU admission after high risk surgery are less likely to remain alive and at home after surgery |
| Vejlgaard *et al*. (2022) | Denmark | Clinical trial | Effectiveness | Examine the impact radical cystectomy has on quality of life comparing robotic-assisted laparoscopic cystectomy with intracorporeal urinary diversion and open radial cystectomy | Patients with bladder cancer eligible for surgery | Robotic-assisted laparoscopic cystectomy with intracorporeal urinary diversion vs. open radial cystectomy | **Days alive and out of hospital**: Incorporated all hospitalizations. Measured at 90 days. | Patients with nausea and vomiting had less DAH. |
| Weigel *et al*. (1999) | Germany | Retrospective cohort study | Descriptive | Evaluate post-operative outcome and quality of life of patients who underwent surgery for symptomatic spinal metastases | Adults who underwent intervention for spinal metastases | N/A | **Time at home:** Definition unclear. Calculated via interviews and medical records, however unclear. | Mean home time was 11.1 months |
| Wu *et al.* (2022) | Australia | Retrospective cohort study | Descriptive | Evaluate DAH within 90 days after hip fracture surgery for older adults | Adults ≥70 years admitted for operative management of hip fracture | N/A | **Days alive and at home:** Incorporated hospitalization, rehabilitation, and days in any facility. Measured at 30 and 90 days. Mortality = 0. | DAH-90 more descriptive in this cohort due to most patients not returning home within 30 days. Several factors associated with reduced DAH as previously shown. |
